# Human mammary cancer progression model recapitulates methylation events associated with breast premalignancy

**DOI:** 10.1186/bcr2457

**Published:** 2009-12-08

**Authors:** Nancy Dumont, Yongping G Crawford, Mahvash Sigaroudinia, Shefali S Nagrani, Matthew B Wilson, Gertrude C Buehring, Gulisa Turashvili, Samuel Aparicio, Mona L Gauthier, Colleen A Fordyce, Kimberly M McDermott, Thea D Tlsty

**Affiliations:** 1 Department of Pathology and Comprehensive Cancer Center, University of California at San Francisco, 513 Parnassus Avenue, San Francisco, California, 94143-0511, USA; 2 Division of Infectious Diseases and Vaccinology, School of Public Health, University of California at Berkeley, Berkeley, California, 94720-7354, USA; 3 Department of Molecular Oncology, B.C. Cancer Research Centre, 675 West 10th Avenue, Vancouver, British Columbia, V5Z 1L3, Canada; 4 Current address: ES Cell Culture, Genentech, Inc. 1 DNA Way, South San Francisco, California, 94080, USA; 5 National Science Foundation, 4201 Wilson Boulevard, Suite 1220, Arlington, Virginia, 22230, USA; 6 Department of Medical Biophysics, University of Toronto, Campbell Family Institute for Breast Cancer Research, 620 University Avenue, Toronto, Ontario, M5G 2C1, Canada; 7 Department of Cell Biology and Anatomy, University of Arizona, 1515 Campbell Avenue, Tucson, Arizona, 85724, USA

## Abstract

**Introduction:**

We have previously identified a rare subpopulation of variant human mammary epithelial cells (vHMEC) with repressed p16^INK4A ^that exist in disease-free women yet display premalignant properties, suggesting that they have engaged the process of malignant transformation. In order to gain insight into the molecular alterations required for vHMEC to progress to malignancy, and to characterize the epigenetic events associated with early progression, we examined the effect of oncogenic stress on the behavior of these cells.

**Methods:**

HMEC that express p16^INK4A ^and vHMEC that do not, were transduced with constitutively active Ha-rasV12 and subsequently exposed to serum to determine whether signals from the cellular microenvironment could cooperate with ras to promote the malignant transformation of vHMEC. Epigenetic alterations were assessed using methylation-specific polymerase chain reaction (PCR).

**Results:**

vHMEC expressing Ha-rasV12 (vHMEC-ras) bypassed the classic proliferative arrest that has been previously documented in normal fibroblasts following oncogenic stress, and that we also observe here in normal HMEC. Moreover, vHMEC-ras cells exhibited many additional alterations that are observed during progression to malignancy such as the generation of chromosomal abnormalities, upregulation of telomerase activity, immortalization following exposure to serum, and anchorage-independent growth, but they did not form tumors following orthotopic injection *in vivo*. Associated with their early progression to malignancy was an increase in the number of genes methylated, two of which (*RASSF1A *and *SFRP1*) were also methylated in other immortalized mammary cell lines as well as in breast cancer cells and tissues.

**Conclusions:**

We have characterized a mammary progression model that recapitulates molecular and methylation alterations observed in many breast cancers. Our data suggest that concomitant methylation of *RASSF1A *and *SFRP1 *marks an early event in mammary transformation and may thus have prognostic potential.

## Introduction

Oncogenic transformation arises from the accumulation of both genetic and epigenetic alterations that result in the activation of oncogenes and inactivation of tumor suppressor genes. Of the many oncogenes activated in human cancers, *ras *is one of the genes that has been the most extensively studied. Although mutation of *ras *genes is rare in human breast cancers [1], over 50% of human breast carcinomas express elevated levels of normal Ha-ras protein [2-4]. In addition, higher levels of ras protein have been observed in hyperplasias from patients who subsequently develop cancer, than in hyperplasias from patients who do not [5]. This suggests that alterations in ras expression can occur early in the transformation process, and thus contribute to the initiation of tumorigenesis. Likewise, epigenetic alterations, including DNA methylation and chromatin structure changes, are among the earliest molecular abnormalities to occur during tumorigenesis. Included among the genes epigenetically silenced in breast cancer are genes involved in cell cycle regulation (*p16*^*INK4A*^, *CCND2, RASSF1A*), cell signaling (*SFRP1, SFRP5*), differentiation (*HOXA9*), immortalization (*p57*), and DNA repair (*MGMT*, *BRCA1*) [6-12]. A recent survey of CpG island methylation using an array-based mapping technique revealed that one-third of CpG islands methylated in premalignant lesions are associated with members of various homeobox gene superfamilies, suggesting that methylation of homeobox genes is a frequent and early event in breast cancer [13].

Consistent with this, we have previously identified a rare subpopulation of variant human mammary epithelial cells (vHMEC) that exhibit *p16*^*INK4A *^and *HOXA9 *promoter hypermethylation, centrosome dysfunction, genomic instability, and COX-2 overexpression [14-17]. We found evidence that cells with these characteristics exist in morphologically normal tissue of disease-free women [18], as well as in ductal carcinoma in situ (DCIS) lesions [19], suggesting that these cells may be precursors to cancer.

In order to gain insight into the molecular alterations required for vHMEC to progress to malignancy, and the epigenetic events associated with that progression, we examined the effect of oncogenic stress on the behavior of HMEC that express p16^INK4A^, and vHMEC that do not, by expressing constitutively active Ha-rasV12 in these cells. Since vHMEC display some characteristics of tumor cells, suggesting that the process of malignant transformation is initiated in these cells, we hypothesized that vHMEC would be resistant to ras-induced growth arrest, but that HMEC, like normal fibroblasts which have been shown to senesce in response to oncogenic ras [20], would not. Indeed, as expected, vHMEC continued to proliferate following ras expression while HMEC arrested. Moreover, when cultured in a serum-containing environment, vHMEC expressing oncogenic ras spontaneously immortalized, acquired the capacity for anchorage-independent growth, and exhibited *de novo *DNA methylation at several gene loci frequently methylated in breast cancer. Among the genes methylated, we identified a panel of four genes, two of which (*RASSF1A *and *SFRP1*), were also methylated in other immortalized mammary cell lines, suggesting that programs of epigenetic alterations mark early steps in the transformation process. Thus, these early epigenetic alterations may prove useful as biomarkers for early detection of breast cancer.

## Materials and methods

### Cell culture and breast tissue specimens

HMEC were isolated from reduction mammoplasty tissue as previously described [21]. HMEC were grown in MEGM (Lonza, Walkersville, MD, USA) supplemented with or without 0.5% fetal bovine serum. Initial experiments examining the effect of oncogenic ras on HMEC and vHMEC proliferation were conducted in cells isolated from five different individuals: RM48 (kindly provided by Martha Stampfer, Lawrence Berkeley National Laboratory, Berkeley, CA), RM13, RM15, RM18, and RM21 (cells derived in our laboratory). All subsequent experiments in which cells were exposed to serum were conducted with RM48. All cell cultures were maintained as previously described [21]. vHMEC clones were isolated using standard ring cloning procedures.

Breast specimens originated from biopsies, mastectomies, and reduction mammoplasties. Donors included both breast cancer patients and women with no breast cancer history. Specimens were acquired as either fresh tissues, frozen tissues, which had been stored at -80°C, or as DNA that had already been extracted from frozen tissues archived in the British Columbia Cancer Agency frozen breast tumor bank and Cooperative Human Tissue Network. Protocols for the acquisition of human specimens were approved by the institutional review boards of the institutions providing the specimens (University of British Columbia, British Columbia Cancer Agency Research Ethics Board, Canada, and the Committee for the Protection of Human Subjects, University of California, Berkeley, CA, USA).

### Retroviral gene transfer

The pLXSP3 and pLXSP3-Ha-rasV12 retroviral constructs were a gift from Dr. Frank McCormick (UCSF Comprehensive Cancer Center, University of California San Francisco, San Francisco, CA, USA). Amphotropic retrovirus was produced by transfecting Phoenix-A packaging cells using Lipofectamine 2000 (Invitrogen, Carlsbad, CA, USA). Forty-eight to 72 hours post-transfection, virus-containing culture medium was collected and filtered through 0.45-μm syringe filters. Cells were infected by exposing them to virus-containing medium in the presence of 4 μg/mL Polybrene (Sigma-Aldrich, St. Louis, MO, USA). Forty-eight hours following infection, transduced cells were selected for four to six days in medium containing 2 μg/mL puromycin. Experiments were conducted four to six days following exposure to puromycin to allow selection of cells that exhibit puromycin resistance.

### Immunoblot analysis

Cells were washed twice with ice-cold phosphate buffered saline (PBS) and lysed with 50 mM Tris, 150 mM NaCl buffer containing 1% Nonidet P-40, 0.25% deoxycholate, 1 mM EDTA, 20 mM sodium fluoride, 1 mM sodium orthovanadate, and 1× cocktail of Complete protease inhibitors (Roche Applied Science, Indianapolis, IN, USA). Protein content was quantitated utilizing the BCA protein assay reagent (Pierce, Rockford, IL, USA). Protein extracts were separated by SDS-PAGE using 4-20% gradient polyacrylamide gels (Cambrex, East Rutherford, NJ, USA), and transferred onto Hybond-P membranes (GE Healthcare Bio, Piscataway, NJ, USA) at 100 volts for two hours. Membranes were blocked with 5% nonfat dry milk in Tris-buffered saline containing Tween (TBS-T) (20 mM Tris-HCl, pH 7.6, 150 mM NaCl, 0.1% Tween 20 (v/v)) for one hour at room temperature and incubated with the primary antibodies diluted in blocking buffer overnight at 4°C. The rabbit polyclonal antibody against Ha-ras (#SC520) was obtained from Santa Cruz Biotechnology (Santa Cruz, CA, USA). The mouse monoclonal antibody against β-actin (#5441) was obtained from Sigma-Aldrich (St. Louis, MO, USA). Following incubation with primary antibodies, the membranes were washed three times for 10 minutes with TBS-T, incubated with horseradish peroxidase-conjugated secondary antibodies for one hour at room temperature, and rewashed three times for 10 minutes with TBS-T. Immunoreactive bands were visualized by chemiluminescence using the SuperSignal West Pico Chemiluminescent Substrate System (Pierce, Rockford, IL, USA).

### Cell cycle analysis

Cells were metabolically labeled with 10 μmol/L bromodeoxyuridine for four hours, trypsinized, and fixed with ice-cold 70% ethanol. Nuclei were isolated and stained with propidium iodide and FITC-conjugated anti-bromodeoxyuridine antibodies (Becton Dickinson, San Jose, CA, USA) and analyzed by flow cytometry using a FACS-Sorter (Becton Dickinson, San Jose, CA, USA) and the CellQuest software (Becton Dickinson, San Jose, CA, USA).

### Chromosomal analysis

Metaphase spreads were prepared from cells treated with 100 ng/ml Colcemid (KaryoMAX, GibcoBRL) for six hours. Standard G-banding karyotypic analysis was performed on at least 50 metaphase spreads for each cell population.

### Centrosome analysis

Centrosomes were stained and analyzed as previously described [17]. Briefly, cells were grown on coverslips, fixed in methanol at -20°C for five minutes and stained using a standard immunocytochemistry protocol with a primary monoclonal antibody that recognizes γ-tubulin (1 μg/mL; clone GTU-88, Sigma) and a secondary fluorescein isothiocyanate (FITC) conjugated sheep F(AB')2 fragment to mouse IgG (whole molecule) (ICN Pharmaceuticals, Inc., Costa Mesa, CA, USA). Samples were analyzed on a Zeiss 510 LSM Confocal Microscope (Carl Zeiss AG, Oberkochen, Germany). Statistical significance was determined by the two-sided Fisher exact test (95% confidence interval).

### Telomerase activity assay

Telomerase activity was measured using the Quantitative Telomerase Detection Kit from Allied Biotech (Vallejo, CA, USA) following the manufacturer's directions. Briefly, 1 × 10^6 ^cells were harvested, snap frozen in liquid nitrogen, and subsequently lysed using the provided lysis buffer. Protein content in the lysates was determined utilizing the BCA protein assay reagent (Pierce, Rockford, IL, USA). The amount of telomerase activity in 1 μg of lysate was compared to the standard provided in the kit. Each sample was analyzed in triplicate. A no template control, heat inactivated sample, and cell lysates from telomerase positive (MDA-MB-231) and telomerase negative (U2OS) cells were included in each experiment.

### Soft agar colony assay

Cells were harvested, counted, and resuspended in media containing 0.6% agarose, 5% serum and 10 mM Hepes. Cell suspensions were then plated on top of a bottom layer of agarose in 35 mm dishes at a concentration of 50,000 cells per dish, in triplicate. After 14 days, colonies were counted manually in eight different fields. The data are presented as the average of the sum of eight different fields counted.

### *In vivo *tumor studies

An IRES-based bicistronic lentiviral vector encoding green fluorescent protein (GFP) and firefly luciferase was obtained from Dr. Sanjiv Sam Gambhir (Stanford University, Palo Alto, CA, USA) and utilized to generate the cells used for the *in vivo *tumor studies. Briefly, virus was produced by co-transfecting 293T cells with the CSCMV-FLuc-EGFP vector along with a defective packaging vector encoding the HIV gal, pol, rev, and tat genes, and a plasmid coding for the VSVG envelope protein. Clones isolated from a pool of immortalized vHMEC-ras0.5 cells were transduced with lentiviral particles. Twenty-four hours following transduction, the cells were split, allowed to expand for one week, and then FACS sorted to collect GFP-expressing cells. Expression of GFP-luciferase did not alter the growth characteristics of these cells (see Additional file 1). One, four, or ten million cells expressing GFP-luciferase were injected directly into the surgically exposed #4 mammary fat pads of six to eight week old female SCID-Beige mice. Cell growth and survival were monitored weekly by bioluminescence imaging utilizing the Xenogen IVIS imaging system (Caliper Life Sciences, Hopkinton, MA, USA). Quantitation of photon emission from the bioluminescent signal was performed utilizing the acquisition and analysis software Living Image (Xenogen). All experiments involving animals were conducted in compliance with the Institutional Animal Care and Use Committee guidelines.

### Methylation-specific PCR

Genomic DNA was isolated from cells using the Wizard Genomic DNA Isolation kit (Promega, Madison, WI, USA). Approximately 750 ng of DNA was bisulfite-treated with the EZ DNA Methylation Gold kit according to the manufacturer's protocol (Zymo Research, Orange, CA, USA). Methylation-specific PCR (MSP) was performed on bisulfite-modified DNA using previously described primer pairs and PCR cycle conditions for *p16*^*INK4A *^[22], *HOXA9 *[16], *CCND2 *[12], *RASSF1A *[23], *SFRP1 *[24], *p57, MGMT *[25], and *THBS1 *[26]. Control templates from human genomic lymphocyte DNA either treated with *SssI *methylase (methylated control) or untreated (unmethylated control) and a no template (water) control were included in each experiment. PCR products were electrophoresed on 3% agarose gels, stained with ethidium bromide, and visualized under UV illumination.

### Real-time quantitative PCR

Total RNA was isolated from cells and cDNA synthesized using standard methods. Quantitative PCR (Taqman, Applied Biosystems, Inc., Foster City, CA, USA) was performed on cDNA using the standard curve method with primer/probe sets (Applied Biosystems, Inc.) for SFRP1 (Hs00610060_m1), RASSF1A (Hs00200394_m1), and MGMT (Hs01037698_m1). The expression of GUSB (IDT), an external control, was used to normalize for variances in input cDNA. The forward and reverse primer sequences for GUSB were: 5'-CTCATTTGGAATTTTGCCGATT-3', 5'-CCGAGGAAGATCCCCTTTTTA-3', 5' FAM-TGAACAGTCACCGACGAGAGTGCTGGTA-TAM 3', respectively. Each experiment was performed at least in triplicate. Error bars represent the standard deviation in a representative experiment.

## Results

### vHMEC are resistant to Ha-ras-induced proliferative arrest and display chromosomal abnormalities

**Figure 1:**
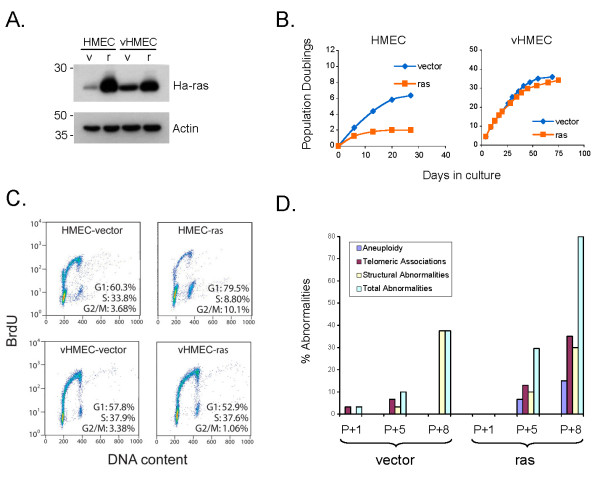
**vHMEC expressing Ha-ras are resistant to proliferative arrest and display increased numbers of chromosomal abnormalities**. **(a)** Immunoblot analysis demonstrating Ha-rasV12 expression in HMEC and vHMEC following retroviral transduction with pLXSP3-Ha-rasV12 (r) or the control pLXSP3 vector (v). Constructs were expressed in HMEC and vHMEC derived from five different individuals. A representative blot is shown along with actin as a loading control. **(b)** Growth curves demonstrating that HMEC underwent a proliferative arrest in response to oncogenic ras (orange line, left graph), while vHMEC continued to proliferate (orange line, right graph). **(c)** Cell cycle analysis of HMEC and vHMEC expressing Ha-rasV12 or control vector demonstrating that the number of cells in S-phase dropped from 33.8% to 8.8% following Ha-rasV12 expression in HMEC, but remained the same in vHMEC (37.9% and 37.6%, respectively). **(d)** Chromosomal analysis of vHMEC-vector and vHMEC-ras cells. Control vHMEC (vector) and vHMEC expressing oncogenic Ha-RasV12 (ras) were harvested at different passages (P+1, P+5, and P+8), as indicated, and processed for metaphase analysis. Standard G-banding karyotypic analysis was performed on at least fifty metaphase spreads for each cell population. Aneuploidy refers to additions or deletions of whole chromosomes. Structural abnormalities include all deletions, duplications, rings, marker chromosomes, chromatid exchanges and translocations. The total number of abnormalities includes all structural abnormalities and telomeric associations, not including numerical abnormalities.

To test the effect of oncogenic stress on normal HMEC and vHMEC with repressed p16^INK4A^, cells were retrovirally transduced with constitutively active Ha-rasV12. Expression of Ha-rasV12 was confirmed by immunoblot analysis (Figure 1a). Despite oncogenic ras expression, vHMEC failed to undergo the classic proliferative arrest that has been previously documented in normal fibroblasts [20], and that we also observe here in normal HMEC (Figure 1b). Instead, they continued to proliferate at the same rate as vHMEC expressing the control vector (vHMEC-vector). The percentage of cells in S phase was not affected by expression of oncogenic ras in vHMEC, but was significantly reduced in HMEC (Figure 1c). These data demonstrate that vHMEC are resistant to Ha-rasV12-induced proliferative arrest.

Progression to malignancy is often associated with an increase in genomic instability. Since vHMEC expressing oncogenic ras (vHMEC-ras) were capable of bypassing the proliferative barrier normally induced by oncogenic stress, we examined whether these cells displayed any alterations in genomic integrity beyond those previously detected in vHMEC [15,17]. This analysis could not be performed in HMEC expressing oncogenic ras since the cells arrested. In vHMEC, no increases in the frequency of centrosome abnormalities were detected between the vHMEC vector control and vHMEC ras-expressing cell populations (data not shown). However, karyotypic analysis revealed a number of chromosomal abnormalities, including structural abnormalities, telomeric associations, and alterations in ploidy (Figure 1d). Thus, upon continued propagation, vHMEC-ras cells become increasingly genomically unstable, manifesting genomic changes at an earlier passage and an increased frequency than detected in cultured vHMEC.

### Signals from the extracellular environment can cooperate with oncogenic ras to immortalize vHMEC and upregulate telomerase activity

**Figure 2:**
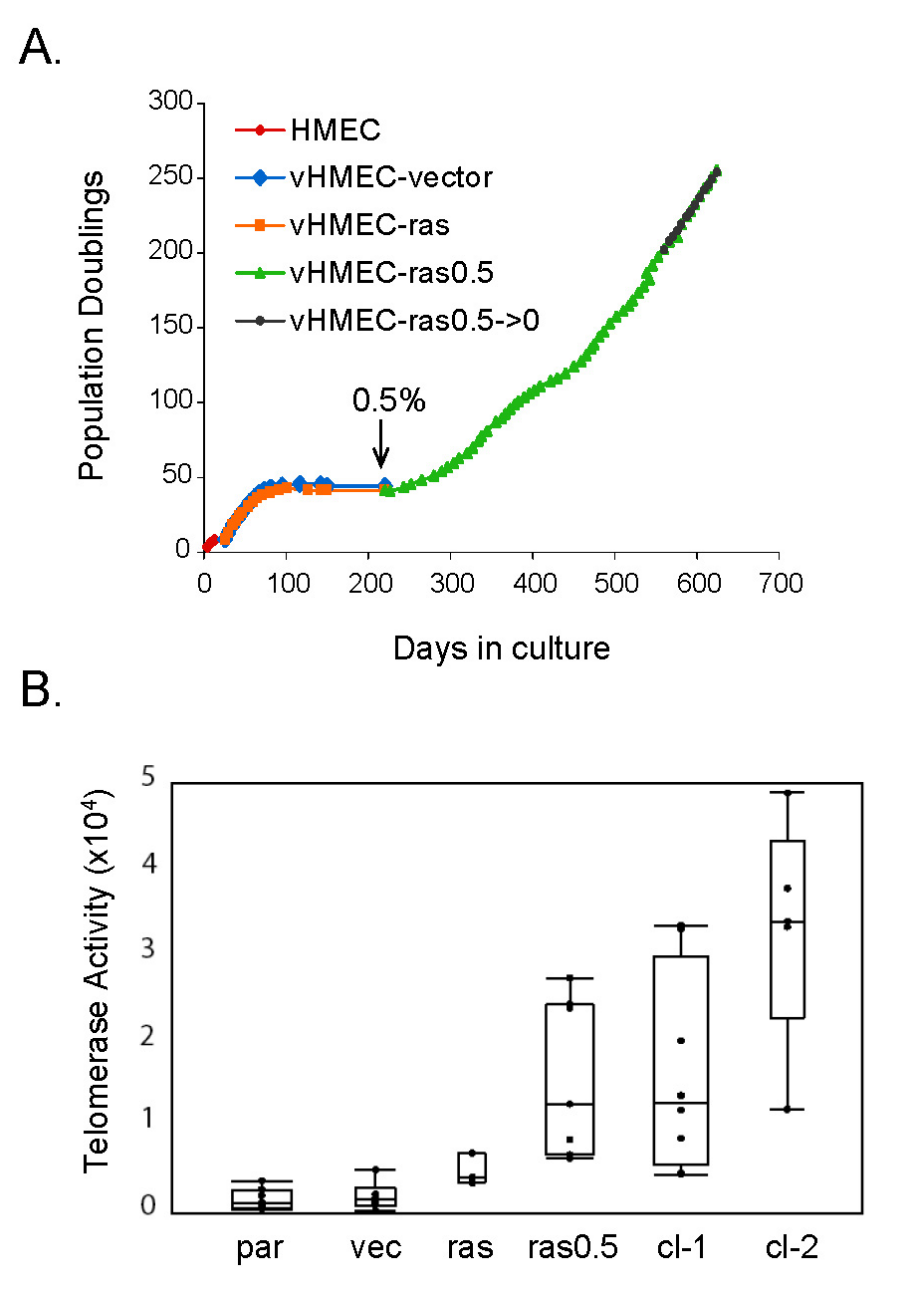
**Signals from the extracellular environment cooperate with ras to immortalize vHMEC and upregulate telomerase activity**. **(a)** Growth curves of HMEC (red line), vHMEC expressing control vector (blue line), and vHMEC expressing Ha-rasV12 (orange line). Arrow indicates time at which vHMEC-ras cells were exposed to serum (day 220). These cells demonstrated increased population doublings within two passages (day 243) in serum-containing media, and are referred to as vHMEC-ras0.5 cells. Their growth curve is depicted in green. After 560 days, the vHMEC-ras0.5 cells were cultured in the absence of serum and continued to proliferate. These cells are referred to as vHMEC-ras0.5- >0 and their growth curve is depicted in black. **(b)** Telomerase activity assay depicting the amount of telomerase activity in lysates prepared from parental vHMEC (par), vHMEC-vector (vec), vHMEC-ras (ras), and vHMEC-ras0.5 (ras0.5) cells, as well as two clones isolated from the ras0.5 cell population (cl-1 and cl-2).

While oncogenic ras can cooperate with a number of viral and cellular genes to transform cells [27,28], whether it can do so in concert with signals from the extracellular milieu has not been examined. Since signals from the microenvironment can promote mammary tumorigenesis [29], we examined whether changes in the cellular environment could influence the effect of ras in vHMEC, and facilitate their progression to malignancy. Studies have shown that the gene expression pattern of cultured primary fibroblasts in response to serum exposure resembles that of a wounding response, and that this wound-response signature is strongly predictive of metastasis and progression for a variety of carcinomas [30]. Therefore, we exposed both vHMEC-vector and vHMEC-ras cells at several points in their growth curve to media containing 0.5% serum. Addition of serum while the cells were still in the exponential growth phase induced a proliferative arrest in both cell populations (data not shown). In contrast, addition of serum while the cells were in a highly mutagenic phase (agonescence) was sufficient to cause immortalization of vHMEC-ras (vHMEC-ras0.5) but not control vHMEC (Figure 2A). This suggests that an additional event, likely conferred by the genomic instability that is exhibited at agonescence (growth plateau) [15], is required for serum to cooperate with ras in immortalizing vHMEC.

To address whether constitutive extracellular stimulation resulting from the exposure to serum was required for the continued proliferation, vHMEC-ras0.5 cells were cultured in mammary epithelial growth medium without serum (vHMEC-ras0.5->0). We found that the cells were capable of continued proliferation in the absence of serum, indicating that once immortalization is initiated by extracellular serum stimulation, proliferation is no longer dependent on the initial signals provided by the serum (Figure 2a). Consistent with this, immortalized vHMEC-ras0.5 cells displayed an increase in telomerase activity relative to the parental, vector control, and vHMEC-ras cells (Figure 2b). In addition, two clones (cl-1 and cl-2) isolated from the vHMEC-ras0.5 cell population also displayed an increase in telomerase activity (Figure 2b).

### Immortalized vHMEC expressing oncogenic ras are capable of anchorage-independent growth but are not tumorigenic *in vivo*

**Figure 3:**
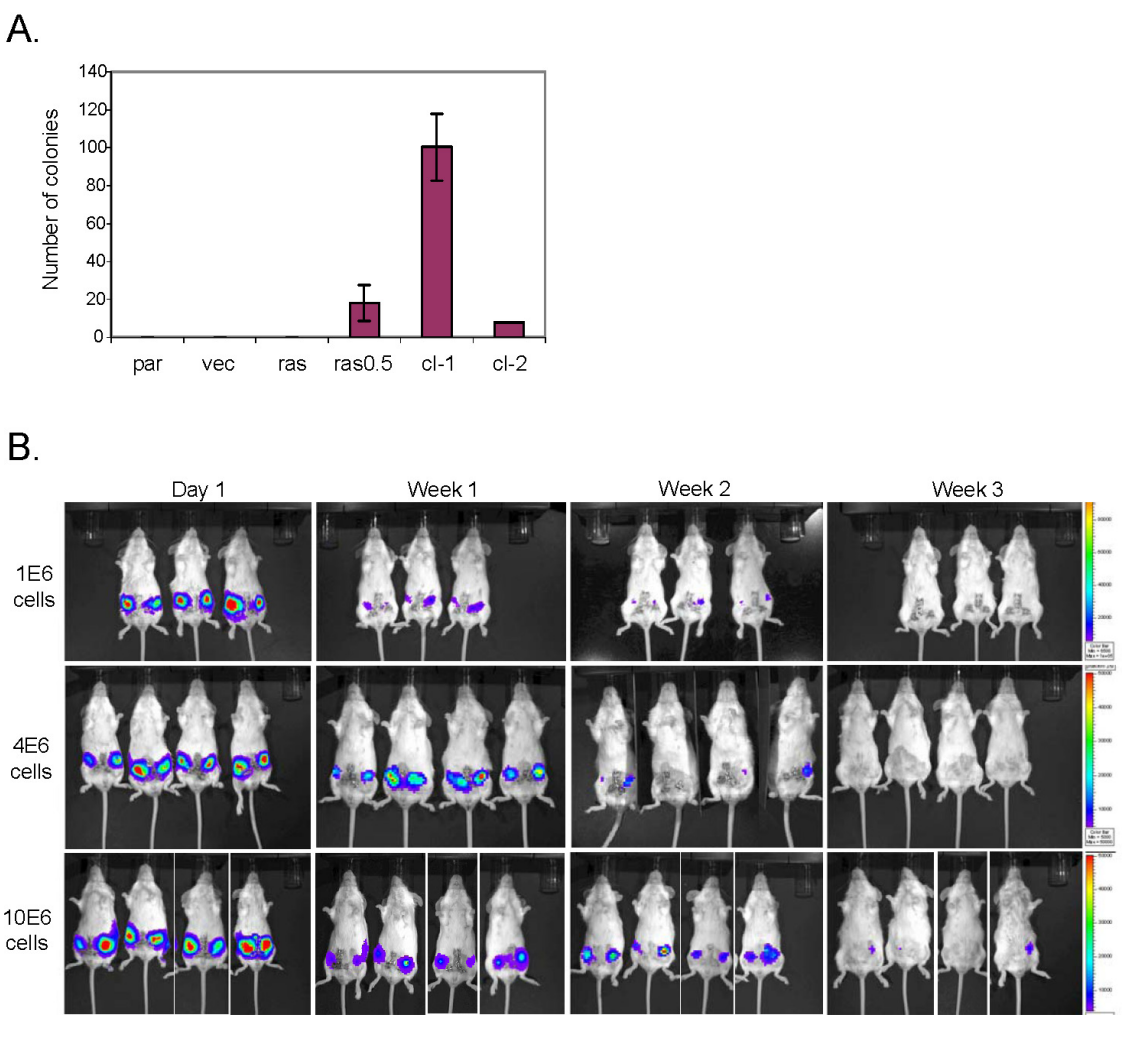
**Immortalized variant human mammary epithelialcells expressing oncogenic ras are capable of anchorage-independent growth but are not tumorigenic *in vivo***. **(a)** Soft agar colony assay. Parental vHMEC (par), vHMEC-vector (vec), vHMEC-ras (ras), vHMEC-ras0.5 (ras0.5), clone 1 (cl-1), and clone 2 (cl-2), which were isolated from the vHMEC-ras0.5 cells, were plated in 35 mm dishes at a concentration of 50,000 cells per dish, in triplicate. After 14 days, colonies were counted manually in eight different fields. The data are presented as the average of the sum of eight different fields counted. **(b)** Bioluminescence imaging of SCID-Beige mice following orthotopic injection of 1 × 10^6 ^(top panel), 4 × 10^6 ^(middle panel), or 10 × 10^6 ^(bottom panel) vHMEC-ras clone 1 cells expressing GFP-luciferase into the left and right #4 mammary fat pad. Cell growth and survival was monitored weekly by bioluminescence imaging utilizing the Xenogen IVIS imaging system. Representative images of each experiment are shown.

Since vHMEC expressing oncogenic ras exhibited many characteristics observed during the progression to malignancy, including increased genomic instability, upregulation of telomerase activity, and unlimited proliferative capacity, we then asked if the accumulation of these molecular alterations had rendered these cells tumorigenic. Since anchorage-independent growth is a requirement for tumorigenicity, we first examined whether vHMEC expressing oncogenic ras could grow in soft agar. As shown in Figure 3a, the parental, vector control, and non-immortalized vHMEC-ras cells failed to grow in soft agar. In contrast, the immortalized vHMEC-ras0.5 as well as two clones isolated from vHMEC-ras0.5 cells (cl-1 and cl-2), displayed some, albeit weak, capacity for anchorage-independent growth, suggesting that they may have some tumorigenic potential.

Since clone 1 exhibited the highest capacity for anchorage-independent growth in the soft agar assay, we decided to examine whether this clone could grow following orthotopic injection into the mammary glands of immunocompromised mice. In order to facilitate the monitoring of its growth *in vivo*, clone 1 was engineered to express firefly luciferase. The light emitted by the clone following administration of the luciferin substrate allowed us to directly visualize the cells in live animals by bioluminescence imaging.

For these experiments, we injected either 1 × 10^6 ^(n = 6), 4 × 10^6 ^(n = 12) or 10 × 10^6 ^(n = 11) clone 1 cells expressing luciferase directly into the surgically exposed #4 mammary fat pads of six to eight week old female Scid/beige mice. In all the glands injected with either 1 × 10^6 ^or 4 × 10^6 ^clone 1 cells, the luminescent signal faded within three weeks following injection, suggesting that the majority of cells were not surviving *in vivo *(Figure 3b). Likewise, in the majority of the glands injected with 10 × 10^6 ^clone 1 cells, the luminescent signal faded within three to four weeks, while in one gland, the bioluminescent signal persisted up to eight weeks (Figure 3b and data not shown). No palpable tumors ever formed in any of these mice. Histological and whole mount analyses of their mammary glands did not reveal any signs of tumor growth or hyperplasia (data not shown). Thus, under the experimental conditions tested, these cells were not tumorigenic, suggesting that additional alterations/mutations are required for complete transformation.

### Progression to malignancy is associated with DNA methylation at several gene loci

**Figure 4:**
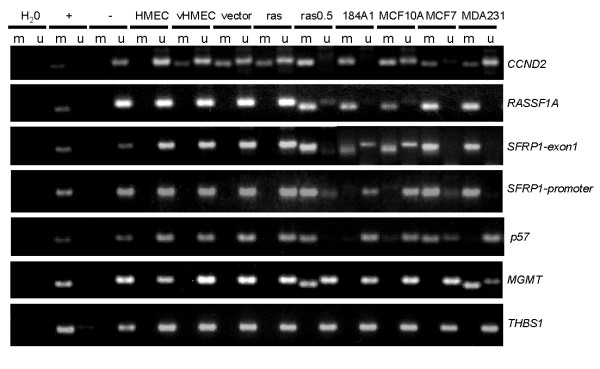
**Progression to malignancy is associated with DNA hypermethylation at several gene loci**. MSP analysis of *CCND2*, *RASSF1A*, *SFRP1, p57, MGMT*, and *THBS1 *in the cells indicated using primer sets listed in Table 1 that specifically amplify either methylated (m) or unmethylated (u) DNA. Positive (+) and (-) controls for the methylated product, as well as a H_2_O negative control, were included in all experiments. All MSP experiments were performed on at least two independent cell populations. The data presented for the *SFRP1-exon1 *MSP are from two different experiments. Analysis of the primary cells (HMEC, vHMEC, vector, ras, and ras0.5) and the cell lines (184A1, MCF10A, MCF7, and MDA231) was done separately and merged. The data presented for all the other genes were obtained in the same experiment. Replicate experiments are available online in Additional file 2.

In order to determine whether the accumulation of alterations observed in our progression model was also accompanied by additional epigenetic alterations, we used methylation-specific PCR (MSP) to determine whether genes that are commonly epigenetically silenced in breast cancer, were also silenced in our cell model. A list of the MSP primers used, along with their location relative to the transcription start site of each gene, is presented in Table 1. Coincident with the *p16*^*INK4A *^and *HOXA9 *promoter hypermethylation we have previously documented in vHMEC [16], we observed methylation of the *CCND2 *promoter in these same cells (Figure 4, Table 2, Additional file 2). As the cells progressed further towards malignancy and became immortalized, DNA methylation was observed at several additional gene loci including, *RASSF1A, SFRP1, p57*, and *MGMT*. In order to determine whether this panel of four genes represented a program of epigenetic alterations important for the immortalization of mammary epithelial cells, we examined whether these genes were methylated in the immortalized, but non-tumorigenic 184-A1 and MCF-10A mammary cells, as well as in the MCF-7 and MDA-231 breast cancer cell lines. DNA methylation of the *RASSF1A *gene, and of one of the CpG islands of the *SFRP1 *gene (SFRP1-exon1), was observed in all four of the cell lines examined, while methylation of the *p57 *gene was observed in two of the four cell lines (MCF-10A and MCF-7), and methylation of the *MGMT *gene was observed only in the MDA-231 cell line (Figure 4, Table 2). The gene encoding thrombospondin (*THBS1*), which is also frequently methylated in breast cancer, remained unmethylated in all the cells. These data suggest that concomitant DNA methylation of the *RASSF1A *and *SFRP1 *(exon 1) genes may play a role in the early steps of mammary transformation.

**Table 1:** MSP primers

Gene Name	Forward Primer (5'-3')	Reverse Primer (5'-3')	Product size (bp)	Primer location relative to TSS (bp)	Ref
*p16INK4A*-U	TTA TTA GAG GGT GGG GTG GAT TGT	CAA CCC CAA ACC ACA ACC ATA A	151	+132 to 283	[22]
*p16INK4A*-M	TTA TTA GAG GGT GGG GCG GAT CGC	GAC CCC GAA CCG CGA CCG TAA	150	+132 to 282	
					
*HOXA9*-U	TTG GGG TTA GAT AGG GAG TTG GGA	AAA AAT AAA AAC AAA AAA CAA ACA AA	167	-4453 to -4286	[16]
*HOXA9*-M	TCG GGG TTA GAT AGG GAG TCG GGA	AAA ATA AAA ACG AAA AAC AAA CGA A	166	-4453 to -4287	
					
*CCND2*-U	AGA GTA TGT GTT AGG GTT GAT T	ACA TCC TCA CCA ACC CTC CA	108	-1167 to -1059	[12]
*CCND2*-M	CGG CGA TTT TAT CGT AGT CG	CTC CAC GCT CGA TCC TTC G	101	-1138 to -1037	
					
*RASSF1A*-U	TTT GGT TGG AGT GTG TTA ATG TG	CAA ACC CCA CAA ACT AAA AAC AA	108	+201 to +309	[23]
*RASSF1A*-M	GTG TTA ACG CGT TGC GTA TC	AAC CCC GCG AAC TAA AAA CGA	94	+213 to +307	
					
*SFRP1*-U*	GTT TTG TAG TTT TTG GAG TTA GTG TTG TGT	CTC AAC CTA CAA TCA AAA ACA ACA CAA ACA	135	-2 to +133	[24]
*SFRP1*-M	TGT AGT TTT CGG AGT TAG TGT CGC GC	CCT ACG ATC GAA AAC GAC GCG AAC G	126	+2 to +128	
					
*SFRP1*-U**	TTT TAG TAA ATT GAA TTT GTT TGT GA	TAA AAT ACA CAA AAC TGG TAG AAC	149	-151 to -2	[40]
*SFRP1*-M	TTT AGT AAA TCG AAT TCG AAT TCG TTC GC	TAA AAT ACG CGA AAC TCC TAC G	148	-150 to -2	
					
*p57*-U	GTT GTT TGT GTT TGT GTA GTT TT	AAA AAT CCC ACA AAC AAC AAA ACA	91	+248 to +339	
*p57*-M	TTG TTC GCG TTT GCG TAG TTT C	AAA TCC CAC GAA CGA CAA AAC G	88	+249 to +337	
					
*MGMT*-U	TTT GTG TTT TGA TGT TTG TAG GTT TTT GT	AAC TCC ACA CTC TTC CAA AAA CAA AAC A	93	+30 to +123	[25]
*MGMT*-M	TTT CGA CGT TCG TAG GTT TTC GC	GCA CTC TTC CGA AAA CGA AAC G	81	+36 to +117	
					
*THBS1*-U	TTG AGT TTG TGT GGT GTA AGA GTA T	CCC CAC TAC CTA ACA CAC AAC T	156	-280 to -124	[26]
*THBS1*-M	GTT CGC GTG GCG TAA GAG TAC	CGC TAC CTA ACG CGC AAC T	149	-276 to -127	

*exon 1

**promoter

**Table 2:** Methylation-specific PCR analysis of genes methylated in the human mammary epithelial cell progression model and in mammary cell lines

	*HMEC*	*vHMEC*	*vector*	*ras*	*ras0.5*	*184-A1*	*MCF-10A*	*MCF-7*	*MDA-231*
***p16* ^*INK4A*^**	U	**M**	**M**	**M**	**M**	**M**	**M**	**M**	**M**

***HOXA9***	U	**M**	**M**	**M**	**M**	**M**	**M**	**M**	**M**
***CCND2***	U	**M**	**M**	**M**	**M**	**M**	**M**	**M**	**M**
***RASSF1A***	U	U	U	U	U/**M**	**M**	**M**	**M**	**M**
SFRP1*	U	U	U	U	**M**	U/**M**	U/**M**	**M**	**M**
***p57***	U	U	U	U	**M**	U	**M**	**M**	U
***MGMT***	U	U	U	U	U/**M**	U	U	U	U/**M**
***THBS1***	U	U	U	U	U	U	U	U	U

SFRP1* Methylation status of the CpG island located in exon 1 is shown.

**Figure 5:**
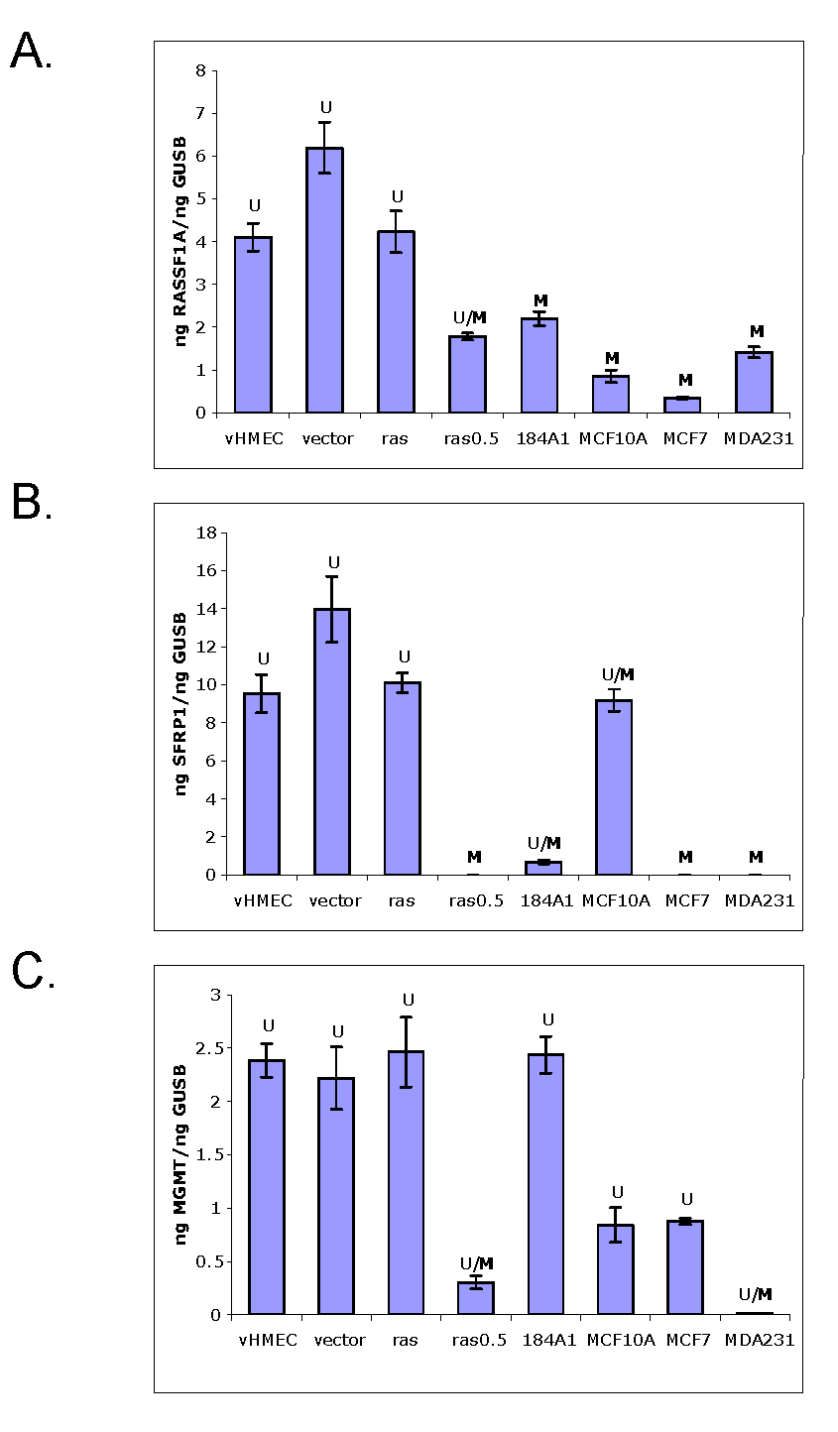
**DNA methylation correlates with reduced gene expression**. Quantitative RT-PCR for **(a)** RASSF1A; **(b)** SFRP1; and **(c)** MGMT. Methylation status as evaluated by MSP is indicated as follows: Unmethylated (U), partially methylated (U/M), or methylated (M). For SFRP1, the U/M labels refer to the methylation status of the CpG island that extends into exon 1.

In order to address this question further, we examined whether methylation of the *RASSF1A *and *SFRP1 *genes led to their silencing. As shown in Figure 5, qPCR analysis revealed that the expression of RASSF1A was reduced in all the cells that exhibited DNA methylation at the *RASSF1A *gene locus. Similarly, SFRP1 expression also correlated with DNA methylation in all the cell lines except the MCF-10A cells, where methylation was observed in the CpG island located in the 3' promoter region that extends into the first exon, but not in the CpG island located in the 5' promoter region (Figure 4), suggesting that methylation in the 5' promoter region may be required for silencing. Upon careful examination, we observed that the degree to which RASSF1A and SFRP1 expression was repressed, corresponded, for the most part, to the extent of DNA methylation, that is, in cells where complete methylation was observed (labeled as "M"), mRNA expression was significantly repressed (except in 184-A1 cells where approximately 50% of RASSF1A expression was retained). In contrast, in cells where both methylated and unmethylated products were observed (labeled as "U/M"), mRNA expression was reduced, but still present. This was also true for MGMT expression, which we examined for comparison (Figure 5c).

### Concomitant DNA methylation of *RASSF1A *and *SFRP1 *occurs in malignant and premalignant breast lesions

**Figure 6:**
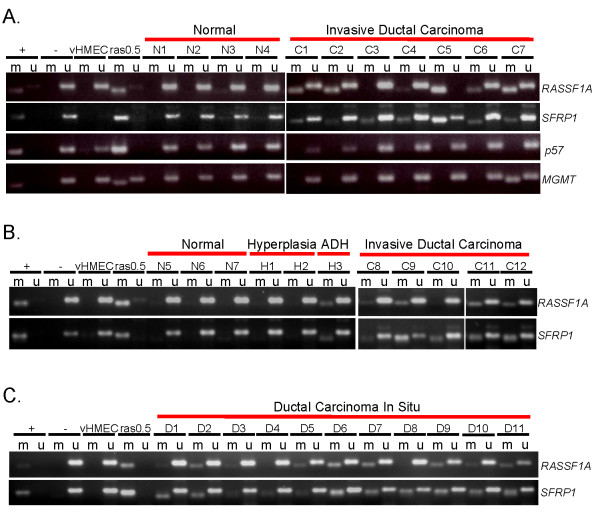
**Concomitant methylation of *RASSF1A *and *SFRP1 *in malignant and premalignant breast tissues**. **(a)** MSP analysis of *RASSF1A*, *SFRP1-exon1, p57*, and *MGMT *in normal and malignant (invasive ductal carcinoma) breast tissues using primer sets that specifically amplify either methylated (m) or unmethylated (u) DNA, as in Figure 4 B and C. MSP analysis of *RASSF1A *and *SFRP1 *in normal, hyperplasia without atypia (H1 and H2), atypical ductal hyperplasia (ADH, H3), invasive ductal carcinoma **(b)**, and ductal carcinomas in situ **(c) **tissues. Additional diagnostic information about these samples is available in Table 3.

If concomitant DNA methylation of the *RASSF1A *and *SFRP1 *genes observed *in vitro *is biologically relevant *in vivo*, we reasoned that DNA methylation of these genes would also be present in breast cancer tissues, but not in normal breast cells or tissues. Therefore, we examined the methylation status of *RASSF1A *and *SFRP1 *in DNA isolated from 12 invasive ductal breast carcinomas (IDC), seven normal breast tissues, and three vHMEC populations isolated from disease-free reduction mammoplasties. As illustrated in Figure 6a and 6b, and summarized in Table 3, this analysis revealed that the *RASSF1A *locus was methylated in 10 out of 12 IDC, none of the vHMECs, and only one of the seven normal tissues (N3, where methylation was very faint). Similarly, the *SFRP1 *locus was methylated in all 12 IDC, none of the vHMECs, and only one of the seven normal tissues. Notably, the normal tissue in which DNA methylation was detected at the *SFRP1 *locus, was also the one in which faint DNA methylation at the *RASSF1A *locus was detected (N3), suggesting that the transformation process may already be initiated in these cells, despite their normal histological appearance. Concomitant methylation of *RASSF1A *and *SFRP1 *appears to be targeted and specific as the *p57 *and *MGMT *gene loci were methylated in none or only one of the IDC analyzed, respectively, and in none of the vHMECs or normal tissues (Figure 6a). These data demonstrate that the concomitant DNA methylation of the *RASSF1A *and *SFRP1 *genes observed upon immortalization of mammary epithelial cells *in vitro*, is biologically relevant in that it can also be detected in both breast cancer cell lines and in breast cancer tissues *in vivo*.

**Table 3:** Description of normal, premalignant, and malignant breast tissues utilized for MSP analysis along with methylation status of *RASSF1A *and *SFRP1 *in each tissue

Sample	Description	RASSF1A	SFRP1
**N1**	Normal reduction mammoplasty tissue	U	U
**N2**	Normal reduction mammoplasty tissue	U	U
**N3**	Normal reduction mammoplasty tissue	**M**	**M**
**N4**	Normal reduction mammoplasty tissue	U	U
**N5**	Normal reduction mammoplasty tissue	U	U
**N6**	Normal reduction mammoplasty tissue	U	U
**N7**	Normal reduction mammoplasty tissue	U	U
**Total Normal** **Methylated**		**1/7**	**1/7**
**H1**	Lobular hyperplasia (85% of mammary cells), blunt duct hyperplasia (15% of mammary cells)	U	U
**H2**	Normal (80%), intraductal hyperplasia (10%), cystic hyperplasia (10%)	U	U
**Total Hyperplasia** **Methylated**		**0/2**	**0/2**
**H3**	Focal atypical terminal duct hyperplasia (single focus); fibrocystic disease	**M**	**M**
**Total ADH** **Methylated**		**1/1**	**1/1**
**D1**	CCL, DCIS	**M**	**M**
**D2**	DCIS	**M**	**M**
**D3**	DCIS, cysts, normal breast tissue	**M**	**M**
**D4**	DCIS and normal tissue	U	**M**
**D5**	Extensive comedo DCIS, calcifications	**M**	**M**
**D6**	Extensive DCIS	**M**	**M**
**D7**	Extensive DCIS and normal tissue	**M**	**M**
**D8**	DCIS, calcifications	U	**M**
**D9**	Extensive DCIS	**M**	**M**
**D10**	DCIS with calcifications	**M**	**M**
**D11**	Extensive comedo DCIS	**M**	**M**
**Total DCIS** **Methylated**		**9/11**	**11/11**
**C1**	IDC, grade 2, node-, ER+	**M**	**M**
**C2**	IDC, grade 3, node+, ER-	**M**	**M**
**C3**	IDC, grade 3, node+, ER-	U	**M**
**C4**	IDC, grade 3, node-, ER-	**M**	**M**
**C5**	IDC, grade 3, node+, ER+	**M**	**M**
**C6**	IDC, grade 1, node+, ER+	**M**	**M**
**C7**	IDC, grade 3, node+, ER-	**M**	**M**
**C8**	IDC, DCIS, CCL, normal breast	**M**	**M**
**C9**	IDC, DCIS	**M**	**M**
**C10**	IDC, DCIS, normal breast	U	**M**
**C11**	IDC and extensive DCIS	**M**	**M**
**C12**	IDC and extensive comedo DCIS with calcifications	**M**	**M**
**Total IDC** **Methylated**		**10/12**	**12/12**

ADH = atypical ductal hyperplasia; CCL = columnar cell lesion; DCIS = ductal carcinoma in situ; IDC = invasive ductal carcinoma; ER = estrogen receptor; U = unmethylated; M = methylated. For *SFRP1*, methylation status of the CpG island located in exon 1 is shown.

In order to determine whether concomitant DNA methylation of the *RASSF1A *and *SFRP1 *genes marks an early event in mammary transformation, we analyzed the methylation status of these two genes in DNA obtained from early lesions. This analysis was performed on two cases of hyperplasia without atypia (H1 and H2), one case of hyperplasia with atypia (H3), and 11 cases of DCIS obtained from women with IDC (see Table 3 for a more detailed description of the samples analyzed). As shown in Figure 6b, DNA methylation was not detected in the hyperplasias without atypia at either gene locus (H1 and H2). In contrast, concomitant DNA methylation of *RASSF1A *and *SFRP1 *was detected in the atypical ductal hyperplasia (H3), which unlike the hyperplasias without atypia, is a premalignant lesion associated with risk for progression to cancer. In the DCIS samples, DNA methylation at the *RASSF1A *locus was observed in 9 of the 11 cases, and methylation of *SFRP1 *was observed in all 11 cases (Figure 6c and Table 3). These data demonstrate that concomitant DNA methylation of *RASSF1A *and *SFRP1 *occurs in premalignant lesions and may mark an early event in mammary transformation.

## Discussion

Malignant progression involves an accumulation of multiple alterations in cellular growth control over an extended period of time. Many studies have shown that oncogenic ras can cooperate with other oncogenic alterations as well as viral oncoproteins to transform primary human cells [28,31]. However, whether oncogenic ras can cooperate with alterations in the extracellular environment to transform primary cells is unknown. Having observed that, unlike HMEC, vHMEC were resistant to the proliferative arrest induced by oncogenic ras and displayed an increase in genomic instability, we sought to determine whether signaling induced by the microenvironment could cooperate with oncogenic stress to transform vHMEC.

In cells isolated from one out of three individuals tested, extracellular stimulation with 0.5% serum at agonescence (which is a highly mutagenic growth plateau), led to the immortalization of vHMEC expressing oncogenic ras. In contrast, stimulation with serum during the exponential phase of growth (prior to agonescence) led to proliferative arrest in these cells. This suggests that an additional mutation, likely conferred by the genomic instability that is exhibited at agonescence [15], is required for serum to cooperate with ras in immortalizing vHMEC.

Altering the cellular microenvironment by simply changing culture conditions can have profound effects on cell behavior. Consistent with this, Shay and colleagues have demonstrated that HMEC expressing the catalytic subunit of telomerase can achieve immortality when grown on fibroblast feeder layers in the presence of 1% serum [32]. In addition, extended cultivation of mammary cells isolated from fibrocystic mammary tissue in growth media containing low concentrations of calcium is what led to the immortalization of the widely used non-tumorigenic MCF-10A mammary epithelial cell line [33]. These data highlight the importance of signals from the microenvironment in modulating cellular behaviors that can influence tumorigenesis.

Interestingly, transient alterations in the cellular environment can permanently alter cell behavior. For example, studies have shown that the non-tumorigenic BPH-1 prostatic epithelial cells can be induced to form tumors in nude mice when recombined with carcinoma-associated fibroblasts or when exposed to carcinogenic doses of steroid hormones. That newly acquired tumorigenicity is maintained in subsequent re-grafting of cells even in the absence of carcinoma-associated fibroblasts or steroid hormones [34]. Likewise, once immortalized, our vHMEC-ras0.5 cells no longer required sustained serum stimulation for continued proliferation, suggesting that signaling induced by serum led to additional alterations sufficient to maintain growth.

Among the alterations we observed in the immortalized vHMEC-ras0.5 cells were an upregulation in telomerase activity as well as DNA methylation at several gene loci, including the *RASSF1A*, *SFRP1*, *p57*, and *MGMT *gene loci. Two of these four genes, *RASSF1A *and *SFRP1*, were also methylated in four other immortalized mammary cell lines we examined, two of which were non-tumorigenic (184-A1 and MCF-10A), and two of which were tumorigenic (MCF-7 and MDA-231). In addition, concomitant DNA methylation of both genes was also observed in malignant (10 out of 12 IDC) and premalignant (1 ADH and 9 out of 11 DCIS) breast lesions. Interestingly, in the reduction mammoplasty tissues in which some hyperplasia without atypia was diagnosed (H1 and H2), no DNA methylation was detected. In contrast, in the reduction mammoplasty tissue with documented atypical ductal hyperplasia (H3), we observed concomitant methylation of *RASSF1A *and *SFRP1 *(Figure 6 and Table 3). While hyperplasias without atypia are not associated with any increased risk for developing breast cancer, atypical ductal hyperplasia (ADH) has been associated with a four- to five-fold increased risk of developing cancer [35], and is thus considered a premalignant lesion. Hence, we have analyzed 12 premalignant lesions (1 ADH and 11 DCIS) and found concomitant methylation of *RASSF1A *and *SFRP1 *in 10 of these 12 samples. Although these data need to be confirmed in a larger sample set, they strongly suggest that concomitant methylation of *RASSF1A *and *SFRP1 *marks an early event in mammary transformation and may thus have diagnostic and/or prognostic potential.

The concomitant methylation of *RASSF1A *and *SFRP1 *also provides possible insights into the biology of early transformation. The *RASSF1A *gene encodes ras association domain family member 1, and the *SFRP1 *gene encodes the Wnt signaling antagonist, secreted frizzled-related protein 1, both of which play an important role in cell proliferation [24,36,37]. Consistent with its role in regulating cell proliferation, *RASSF1A *has been reported to be the most frequently methylated gene in SV40- and hTERT-immortalized prostate epithelial cells [38]. Similarly, we have observed that *RASSF1A *is methylated in the SV40-immortalized human skin fibroblast cell line, GM847 (data not shown). These data suggest that epigenetic alterations in *RASSF1A *occur early in several cell types, not just mammary cells. This is further supported by the fact that *RASSF1A*-deficient mice display increased susceptibility to spontaneous tumor development in multiple organs, including breast, lung, and skin [39]. Compelling evidence suggests that activation of Wnt signaling plays an important role in breast cancer, and that loss of SFRP1 function is a key mechanism by which Wnt signaling is activated under such circumstances [24]. Like *RASSF1A*, *SFRP1 *has also been shown to be silenced through promoter methylation in breast cancer [12,24,40]. We now observe concomitant methylation of *RASSF1A *and *SFRP1 *in three immortalized but non-tumorigenic mammary cell lines as well as in premalignant breast lesions (ADH and DCIS), suggesting that these epigenetic alterations occur early in the transformation process.

Interestingly, we observed a correlation between *SFRP1 *DNA methylation and repression of its expression in all the cell lines we examined except the MCF-10A cells where both methylated and unmethylated DNA products were detected by MSP. While moderate levels of gene expression are expected under circumstances where only partial methylation is observed at a gene locus, the robust expression of SFRP1 in MCF-10A cells was not anticipated. Although we detected DNA methylation in the first exon of *SFRP1 *in MCF-10A cells, previous studies by Lo et al., have demonstrated that the promoter region of *SFRP1 *is unmethylated in these cells [40]. We therefore performed additional experiments with MSP primers designed to survey the methylation status of CpG islands located in the promoter region of *SFRP1*, as described in Lo et al., and confirmed the unmethylated status in that specific region (Figure 4). This supports the well established concept that the location of the CpG dinucleotides subjected to methylation plays an important role in determining whether genes are silenced or not. Consistent with this, *CST6*, which encodes the cysteine protease inhibitor, cystatin M, has been shown to be silenced following DNA methylation of CpG islands located within the promoter region, but not within exon 1 [41]. Of particular note, in MCF-7 and MDA-231 cells where SFRP1 expression is completely abrogated, we observed complete methylation in CpG islands located in the first exon and in the promoter region of *SFRP1 *in these cells. Interestingly, the frequency of DNA methylation in the promoter region and first exon seems to differ. While we observed DNA methylation in exon 1 of the *SFRP1 *gene in 100% of the DCIS and IDC samples we examined, methylation in the promoter region of *SFRP1 *has been reported to occur in approximately 68% of high-grade DCIS and invasive breast cancers [7,40]. These data suggest that there may be different functional consequences to DNA methylation at different CpG islands and that methylation events within a single gene can occur at different frequencies.

While DNA methylation is often associated with gene silencing, methylation marks that are not associated with gene silencing are nonetheless significant. For example, promoter methylation of *TWIST1*, an anti-apoptotic and pro-metastatic transcription factor, is significantly more prevalent in malignant breast tissue than in healthy breast tissue [42], and even more prevalent in metastatic lesions relative to matched primary cancers [43]. However, there is no correlation between *TWIST1 *promoter methylation and TWIST1 mRNA or protein expression [42]. Methylation of the *TWIST1 *promoter may lead to the recruitment of chromatin remodeling proteins that can alter the function and/or expression of neighboring genes. Alternatively, TWIST1 expression may be regulated by a balance between promoter and intragenic CpG methylation as there is evidence that intragenic CpG methylation can promote gene expression [44]. Thus, even though TWIST1 expression is not repressed by methylation, the compelling association between *TWIST1 *CpG methylation and malignancy makes it a useful biomarker for breast cancer diagnosis and prognosis. Likewise, *SFRP1 *methylation in the first exon may be of significance despite the lack of methylation in the 5' promoter region, and the absence of gene silencing. In support of this, recent studies have demonstrated that more than half of the methylated CpG islands in normal genomes fall within the body of the genes or in downstream regions [45]. In addition, numerous CpG islands mapping to intragenic or downstream regions of genes have been reported to be heavily methylated in DCIS, highlighting the significance of intragenic CpG island methylation in the early stages of breast cancer [13].

## Conclusions

Cells without p16 function are resistant to arrest induced by ras-associated oncogenic stress. The accelerated accumulation of genetic and epigenetic events dictate their ability to bypass additional arrest signals allowing rare emergent subpopulations to immortalize, and grow in soft agar. We have identified a multigene methylation pattern acquired during this *in vitro *progression to malignancy that is detected *in vivo *in both premalignant and malignant lesions. This model will thus allow further study of the mechanisms underlying the accumulation of epigenetic alterations that occur during progression to malignancy. By characterizing the methylation profiles that manifest at different stages of transformation, biomarkers with diagnostic and/or prognostic value could eventually be identified.

## Abbreviations

ADH: atypical ductal hyperplasia; BCA: bicinchoninic acid; CCL: columnar cell lesion; DCIS: ductal carcinoma in situ; ER: estrogen receptor; FITC: fluorescein isothiocyanate; GFP: green fluorescent protein; HMEC: human mammary epithelial cells; IDC: invasive ductal carcinoma; MSP: methylation-specific PCR; PBS: phosphate-buffered saline; TBS-T: tris-buffered saline containing tween; vHMEC: variant HMEC with repressed p16^INK4A^.

## Competing interests

The authors declare that they have no competing interests.

## Authors' contributions

ND participated in the design of the studies, performed the immunoblot analysis, the serum-free cell culture experiments, the soft agar assays, the *in vivo *tumor studies, the bioluminescence imaging analysis, part of the MSP experiments, and wrote the manuscript. YGC generated the vHMEC cells expressing oncogenic ras and exposed them to serum. MBW initiated the MSP experiments, which were repeated and completed by MS and ND. SN performed the qPCR experiments, mapped all the MSP primers, and generated Table 1. GB, GT, and SA provided the premalignant and malignant tissue DNA samples. MLG generated the HMEC expressing oncogenic ras and performed the BrDU analysis. CAF performed the telomerase assay. KMM performed the centrosome analysis. TDT contributed to the conception and design of the experiments, and the critical revision of the manuscript. All authors read and approved the final manuscript.

## Supplementary Material

Additional file 1 A PowerPoint file containing a figure illustrating the growth curves of vHMEC-ras0.5 clone 1 cells and vHMEC-ras0.5 clone 1 cells expressing GFP and luciferase (clone 1 GFP-Lux), which indicates that expression of GFP and luciferase does not alter the growth characteristics of these cells. Click here for file

Additional file 2 A PowerPoint file containing a figure illustrating MSP analysis of *RASSF1A *and *SFRP1 *in vHMEC, ras0.5, and two different preparations of 184A1 cells, which demonstrates that both genes are methylated in ras0.5 and 184A1 cells, but unmethylated in vHMEC. The experiments were conducted using primer sets listed in Table 1 that specifically amplify either methylated (m) or unmethylated (u) DNA. Positive (+) and (-) controls for the methylated product, as well as a H_2_O negative control are shown. Click here for file
