# Supplementary figures and images for: Human mammary cancer progression model recapitulates methylation events associated with breast premalignancy
Source: Breast Cancer Res. 2009 Dec 8;11(6):R87. doi: 10.1186/bcr2457 (PMC2815551; doi:10.1186/bcr2457)

## Slide 1
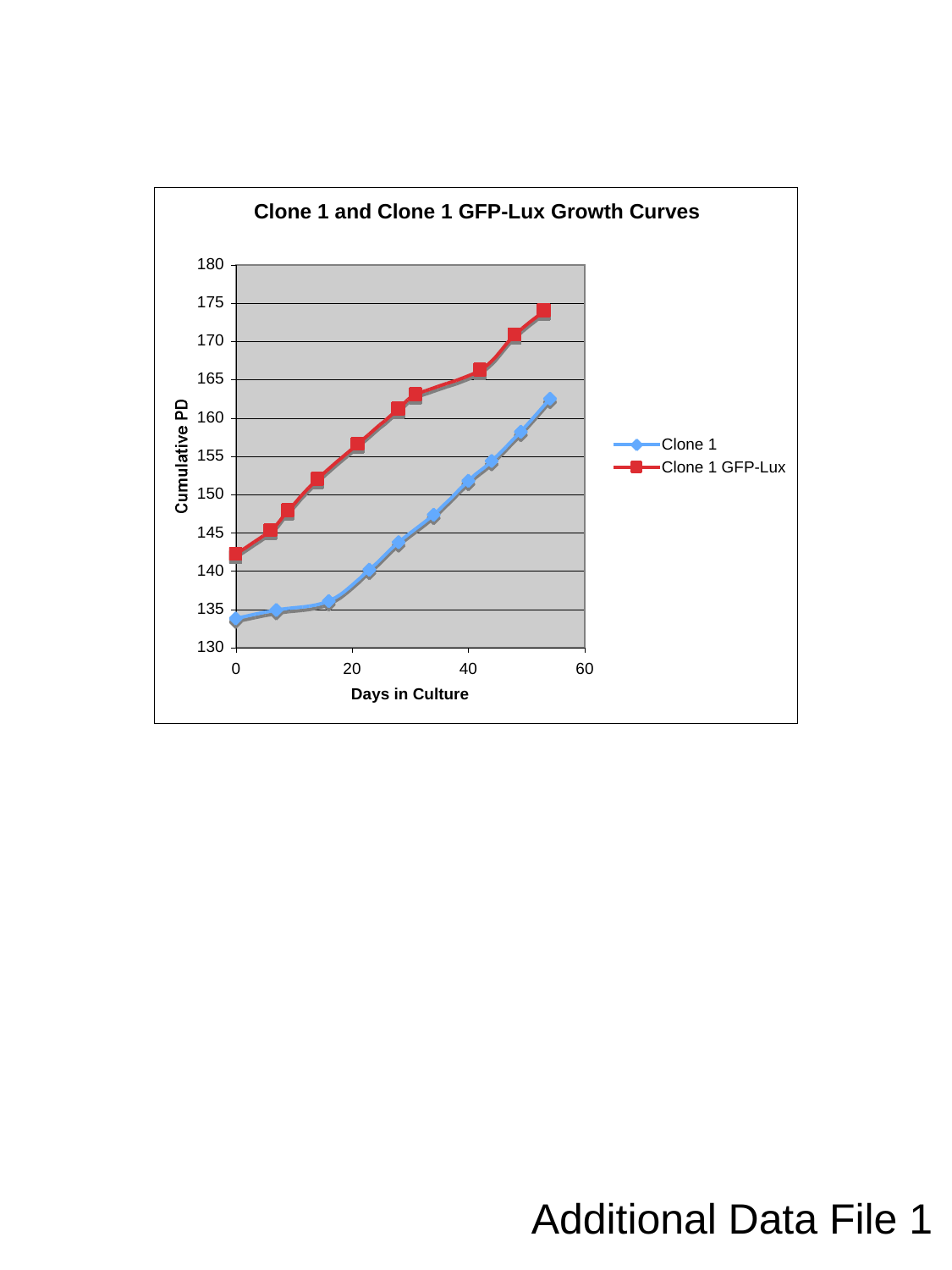

Additional Data File 1

Supplement: Additional file 1 — A PowerPoint file containing a figure illustrating the growth curves of vHMEC-ras0.5 clone 1 cells and vHMEC-ras0.5 clone 1 cells expressing GFP and luciferase (clone 1 GFP-Lux), which indicates that expression of GFP and luciferase does not alter the growth characteristics of these cells. [file bcr2457-S1.ppt]

## Slide 1
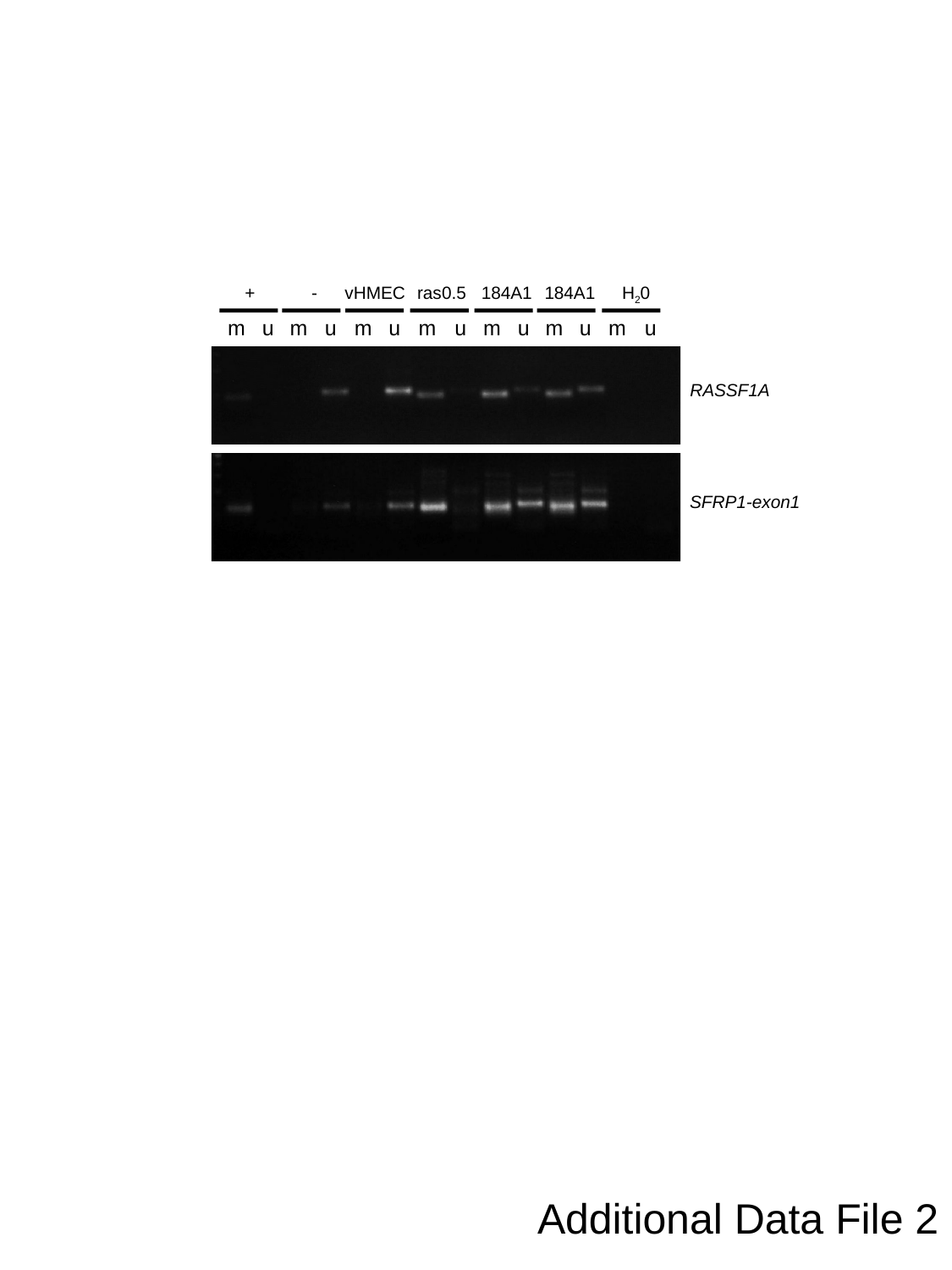

+
-
vHMEC
ras0.5
184A1
184A1
H20
m
u
m
u
m
u
m
u
m
u
m
u
m
u
RASSF1A
SFRP1-exon1
Additional Data File 2

Supplement: Additional file 2 — A PowerPoint file containing a figure illustrating MSP analysis of RASSF1A and SFRP1 in vHMEC, ras0.5, and two different preparations of 184A1 cells, which demonstrates that both genes are methylated in ras0.5 and 184A1 cells, but unmethylated in vHMEC. The experiments were conducted using primer sets listed in Table 1 that specifically amplify either methylated (m) or unmethylated (u) DNA. Positive (+) and (-) controls for the methylated product, as well as a H2O negative control are shown. [file bcr2457-S2.ppt]
